# Crude-oil degradation capabilities by microscopic fungi of deep-sea hydrothermal vents

**DOI:** 10.1007/s11356-025-36879-2

**Published:** 2025-08-29

**Authors:** Diana L. Salcedo, Patricia Velez, Simón López-Ramírez, Rogelio Quiñones-Martínez, Fernando Barragán-Aroche, Luis A. Soto, Mario Figueroa

**Affiliations:** 1 Departamento de Botánica, Instituto de Biología, Universidad Nacional Autónoma de México, Mexico City, 04510 Mexico; 2 Unidad de Servicios para la Industria Petrolera, Facultad de Química, Universidad Nacional Autónoma de México, Mexico City, 04510 Mexico; 3 Departamento de Ingeniería Petrolera, Facultad de Ingeniería, Universidad Nacional Autónoma de México, Mexico City, 04510 Mexico; 4 Instituto de Ciencias del Mar y Limnología, Universidad Nacional Autónoma de México, Coyoacán, Mexico City, 04510 Mexico; 5 Departamento de Farmacia, Facultad de Química, Universidad Nacional Autónoma de México, Mexico City, 04510 Mexico

**Keywords:** Bioremediation, Hydrocarbon pollution, Hydrothermal Vents, Microscopic fungi, Petroleum degradation, Stable isotopes

## Abstract

Selected microscopic fungi from extreme marine ecosystems have unique capacities to degrade complex oil molecules, which confers them a growing interest in bioremediation. This study evaluated the oil-degrading capabilities of six fungal isolates from deep-sea hydrothermal vents. The response of the isolates to the presence of light crude oil (LCO) and heavy crude oil (HCO) was assessed through a tolerance bioassay, while their capabilities to degrade the oil as the sole carbon source were tested in a biodegradation bioassay. The assimilation of oil derivatives into fungal tissues was determined by their carbon (*δ*^13^C) and nitrogen (*δ*^15^N) isotopes. The compositional changes in HCO after exposure to *Aspergillus terreus* and *Penicillium miczynskii* (isolate F) were assessed. All isolates grew in the presence of both oils in the tolerance bioassays, yet *A. terreus*, *Aspergillus sydowii*, and *P. miczynskii* (isolates E and F) showed enhanced growth when using crude oil as the sole carbon source. The *δ*^13^C was more enriched in the isolates than in the oil, suggesting they used it as a carbon source. The analysis of the oil exposed to fungal activity showed that fungi degraded medium and long-chain hydrocarbons. Particularily, *P. miczynskii* (F) showed a remarkable growth in the bioassays and the ability to degrade complex hydrocarbons, representing a promising bioremediation agent.

## Introduction

The increased demand for oil worldwide and the accelerated exploitation of fossil fuels have generated significant environmental impacts. The exploitation, production, refining, and transportation of oil and its derivatives occasionally entail technical and operational accidents, which can lead to massive spills (León et al. 2009; Pernía et al. 2012; Pu 2017). Oil spills are a devastating environmental disaster causing severe impacts on the ocean ecosystems. Coastal habitats, open waters, and the seabed are vulnerable to oil contamination due to the persistence of toxic compounds and their incorporation into food webs or their deposition in sediments (Chang et al. 2014; Pu 2016; Soto et al. 2018).

Due to its geological origin, the Gulf of Mexico (GoM) is an ideal semi-closed basin for accumulating fossil oil and gas deposits (Núñez-Useche et al. 2009). This attribute has historically exposed the GoM to the natural emanation of oil and gas from the seabed, which has been recorded in various sectors of the area and is an essential source of contamination (Wilson et al. 1974; MacDonald et al. 2015). The stability and resilience of the GoM have recently been tested by anthropogenic disturbances (Soto et al. 2018). Two massive crude oil spills have occurred in this ecosystem. One was due to the lack of control of the Ixtoc-I well in the Sonda de Campeche in 1979, and the other was due to the explosion of the Deepwater Horizon (DWH) oil platform off the coast of Louisiana in 2010. These catastrophes have caused severe damage to the environmental health and the communities of the GoM (Sammarco et al. 2013; Soto et al. 2014, 2017, 2018; Salcedo et al. 2017). Additionally, the long-term toxic effects derived from the persistence of hydrocarbons are still a serious threat to the Mexican fishing industry (Soto et al. 2017).

Physical and chemical mitigation engineering methods play an essential role after oil spills. However, current remedial techniques are ineffective and sometimes even more damaging than the oil itself (Chang et al. 2014; Pu 2017). For this reason, a sustainable oil industry requires implementing efficient, economical, and environmentally friendly methods allowing the rehabilitation of ecosystems altered by anthropogenic actions (Zhang and Liyun 2011; Naranjo-Briceño et al. 2019). One such strategy is bioremediation, a process in which living organisms remove, neutralize, or convert environmental contaminants into less harmful products (Mueller et al. 1996; Vidali 2001; Silva et al. 2015). Microscopic organisms such as fungi, algae, and bacteria are often used for that purpose (Johnsen et al. 2005). This low-cost alternative avoids secondary contamination during oil spill cleanup since it contributes to the complete mineralization of hydrocarbons into carbon dioxide and water or may convert them into low-toxic or nontoxic materials (Johnsen et al. 2005; Fuentes et al. 2014; Wang et al. 2016).

Microscopic fungi from extreme ecosystems, such as deep-sea hydrothermal vents, may have unique biodegradation capabilities (Cerniglia and Sutherland 2010). These microorganisms have evolved to cope with extreme environmental conditions, involving new physiological characteristics (e.g. changes in their metabolic pathways) that may be helpful in biotechnological applications such as bioremediation (Rampelotto 2013). Nonetheless, they remain comparatively underexplored in comparison with further members of the microbial communities such as bacteria (Nagano and Nagahama 2012; Nawaz et al. 2022).

Fungi can perform bioremediation through different processes. Despite their low biodegradability, they can use hydrocarbons as carbon and energy sources (Arulazhagan et al. 2017). They also transform insoluble hydrocarbons into soluble compounds using extracellular oxidative enzymes (laccases and peroxidases), which makes them highly efficient in degrading crude oil into lighter fractions (Foght 2004; Harms et al. 2011; Zhang et al. 2016). Fungi have a wide diversity of enzymatic mechanisms that transform the chemical structure of hydrocarbons, both short-chain aliphatic and high molecular weight polycyclic aromatic (Prenafeta-Boldú et al. 2018). Some species absorb hydrocarbons and bioaccumulate them in lipid vesicles (Abruscia et al. 2007; Wu et al. 2008; Li et al. 2010). Moreover, some fungi are recognized for their capabilities to produce biosurfactants (Kiran et al. 2009). Particularly, ligninolytic enzymes from deep-sea derived fungi have robust properties, such as thermostability and halotolerance; in addition, they have been reported to operate under anoxic conditions (Batista-García et al. 2017), representing a promising alternative for bioremediation.

In Mexico, some authors have tested the capabilities of deep-sea isolates from the GoM to degrade hexadecane and 1-hexadecane (Velez et al. 2020), heavy crude oil (HCO) and extra-heavy crude oil (Romero-Hernández et al. 2021). However, little is known about the metabolic capacities of hydrothermal vent fungi to degrade hydrocarbons. Therefore, generating information in this regard is essential to lay the foundations for the biotechnological use of these organisms as potential bioremediation agents to face accidental oil spills. Hence, this research aimed to assess the capabilities of six fungal strains isolated from deep-sea hydrothermal vents to metabolize crude oil as the sole carbon source and to evaluate the potential changes in the oil composition induced by fungal activity. This research represents a novel contribution to understanding the bioremediation potential of deep-sea hydrothermal vent fungi, opening opportunities for innovative environmental solutions and targeted enzymatic strategies for mitigating marine oil spills.

## Materials and methods

### Biological material

Six fungal isolates were retrieved from the Culture Collection of the Laboratorio de Ecología Molecular de Micromicetes en Ecosistemas Amenazados, C202 of the Institute of Biology, UNAM, Mexico (*A. terreus*, *A. sydowii*, *Aspergillus* sp., *Penicillium miczynskii* (isolate E), *Penicillium miczynskii* (isolate F), and *Penicillium dodgei*). These strains were isolated by Velez et al. (2025) from superficial sediments of the Pescadero Basin, Pescadero Transform Fault, and Alarcón Rise hydrothermal vents. They were identified based on the ITS1-5.8S-ITS4 region and beta-tubulin gene sequences (Velez et al. 2025). The sediments from which the fungi were isolated were sampled in deep-sea hydrothermal vents of the southern Gulf of California by the first author through a collaboration agreement with the Monterey Bay Aquarium Research Institute (MBARI).

### Tolerance bioassay

Two different types of crude oil were employed in the bioassays: light crude oil (LCO) extracted from a marine oil well located in the southern Gulf of Mexico and heavy crude oil (HCO) (12 API) obtained from an inland well in Tabasco, a southeastern region of Mexico. To evaluate the tolerance of the fungi to the presence of crude oil, agar plugs of 5 × 5 mm of the six isolates were taken from the margin of the actively growing colonies and inoculated in plates containing solid media prepared with Vogel´s minimal medium (50X Salt Solution 1%; VBE; Moltox), sucrose (1.5%), and 5% of crude oil. The 5% crude oil concentration was selected based on previous studies demonstrating that efficient microbial decomposition of petroleum hydrocarbons occurs at a relatively low initial content of oil (up to 5%) (Harms et al. 2011; Sari et al. 2019; Galitskaya et al. 2021).

The experiments with LCO and HCO were conducted separately, allowing each treatment to have its own control. However, in the case of *P. miczynskii* (F), only one control was used for the growth graphs since the other was contaminated. The Petri dishes were incubated under laboratory temperature (23°C) and dark conditions for 21 days. Cultures devoid of oil served as controls. All the experiments were set up in triplicate. The experiments were photo-documented with a camera Nikon D750 on days 1, 3, 5, 7, 9, 11, 13, 15, and 21. The images were analyzed through ImageJ (Schneider et al. 2012) to determine the radial growth over time. This practical approach allowed the approximation of fungal growth (increase in colony diameter on solid media; Hendricks et al. 2017) to conduct an initial screening of their performance in presence of the tested hydrocarbons.

### Biodegradation bioassay

The capabilities of the isolates to degrade crude oil as the sole carbon source were tested in a biodegradation bioassay. The fungal mycelia were inoculated in flasks containing 10 ml of liquid Vogel´s Minimal Medium (50X Salt Solution, Moltox, at 1%) supplemented with 1% of two different types of petroleum (LCO and HCO) as the sole carbon source (Asemoloye et al. 2020). The cultures were incubated under laboratory temperature (23°C) and dark conditions on a rotatory shaker at 180 rpm for 8 h per day for a month. Inoculated media without oil served as controls. The experiment was conducted in triplicates. At the end of the bioassay, 10 ml of toluene was added to the cultures to separate the mycelium from the crude oil. Next, the flasks were centrifuged at 8,000 rpm for 15 min, and the upper layer containing the remaining oil was removed. This procedure was repeated three times until the oil residues were removed entirely. The layer containing the mycelium was filtered in a Büchner funnel attached to a vacuum pump through wwPTFE membrane disc filters, 47 mm diameter and 0.45 µm pore size (Pall Corporation). The fungal biomass was dried at 36 °C for 24 h. The filters were weighed before and after to obtain the dry-weight biomass of the isolates. The dried biomass was stored for further analysis. It is worth mentioning that the toluene was not added to the samples designated for the compositional analysis of the oil.

### Isotopic analysis

The carbon (*δ*^13^C) and nitrogen (*δ*^15^N) isotopic signatures of the oil samples and isolates were determined and compared to evaluate the assimilation of oil derivatives into fungal tissues (Griffith 2004; Mayor et al. 2009). The dehydrated mycelia obtained from the biodegradation bioassay were stored in Eppendorf tubes and sent to the Stable Isotope Ratio Facility for Environmental Research (SIRFER), University of Utah, for their isotopic determination. The isotopic analysis of the crude oil samples was conducted in the Laboratorio de Análisis de Isótopos Estables (LAIE), UNAM, Yucatán. Values of *δ*^13^C are reported relative to the Vienna Peedee Belemnite (VPDB) scale, and *δ*^15^N values relative to the AIR scale. Delta notation as parts per thousand (‰) was employed. Each sample was run in triplicate, and values were averaged. The isotopic fractionation was determined as the difference in *δ*^13^C and *δ*^15^N values between the product and substrate, in this case, between the dry fungal biomass and the oil.

### Crude oil compositional analysis

The compositional analysis of liquid oil samples, before and after their exposure to fungal activity, was conducted on a gas chromatograph (Agilent, 7890B) equipped with a 30 m capillary column (Agilent, J&W EZ-Guard VF-1ms) and a flame ionization detector (GC-FID). Helium (99.999%) was used as the carrier gas. The detection range covers C3 to C29 and lumped C30 +.

The samples were placed in plastic tubes and carefully separated into aqueous and oil phases using a syringe. The oil phase was diluted with toluene (HPLC grade) at 1:4. Finally, 0.01 g of 1-hexene (> 99%) was added as a reference. The system was vigorously agitated to inject a homogeneous solution into the gas chromatograph. Direct injection of a liquid oil-toluene solution is a simplified and efficient GC method for compositional analysis (Wang et al. 2014). This method ensures the components analysis of the HCO since all its hydrocarbon components are soluble in toluene, and asphaltene aggregation does not occur (Beeka et al. 2018; Fayazi 2019). The amount of used toluene was eliminated in the final percentage mol reported in this work. It is worth mentioning that the LCO exposed to fungal activity could not be analyzed since the remaining amount after the experiment was insufficient for direct GC analysis, so larger culture volumes are recommended for future investigations.

### Statistical analysis

A permutational multivariate analysis of variance PERMANOVA was conducted to analyze the mycelia growth between controls and experiments containing oil in the tolerance and biodegradation bioassays. In the tolerance bioassay, the analysis was carried out on growth on mm^2^, while in the biodegradation bioassay on mg dry biomass. A third PERMANOVA analysis was carried out on the isotopic values of carbon and nitrogen. The analysis was conducted on average values and based on a two-way model including TREATMENT (3 levels) and ISOLATES (6 levels) as fixed factors, using 9999 permutations under the reduced model. Considering only factors showing statistical differences in the previous analysis, a pairwise test was performed to analyze specific differences between their levels. All statistical analyses were conducted in the PRIMER v6 and PERMANOVA add-on statistical software.

## Results

### Tolerance bioassay

**Fig. 1:**
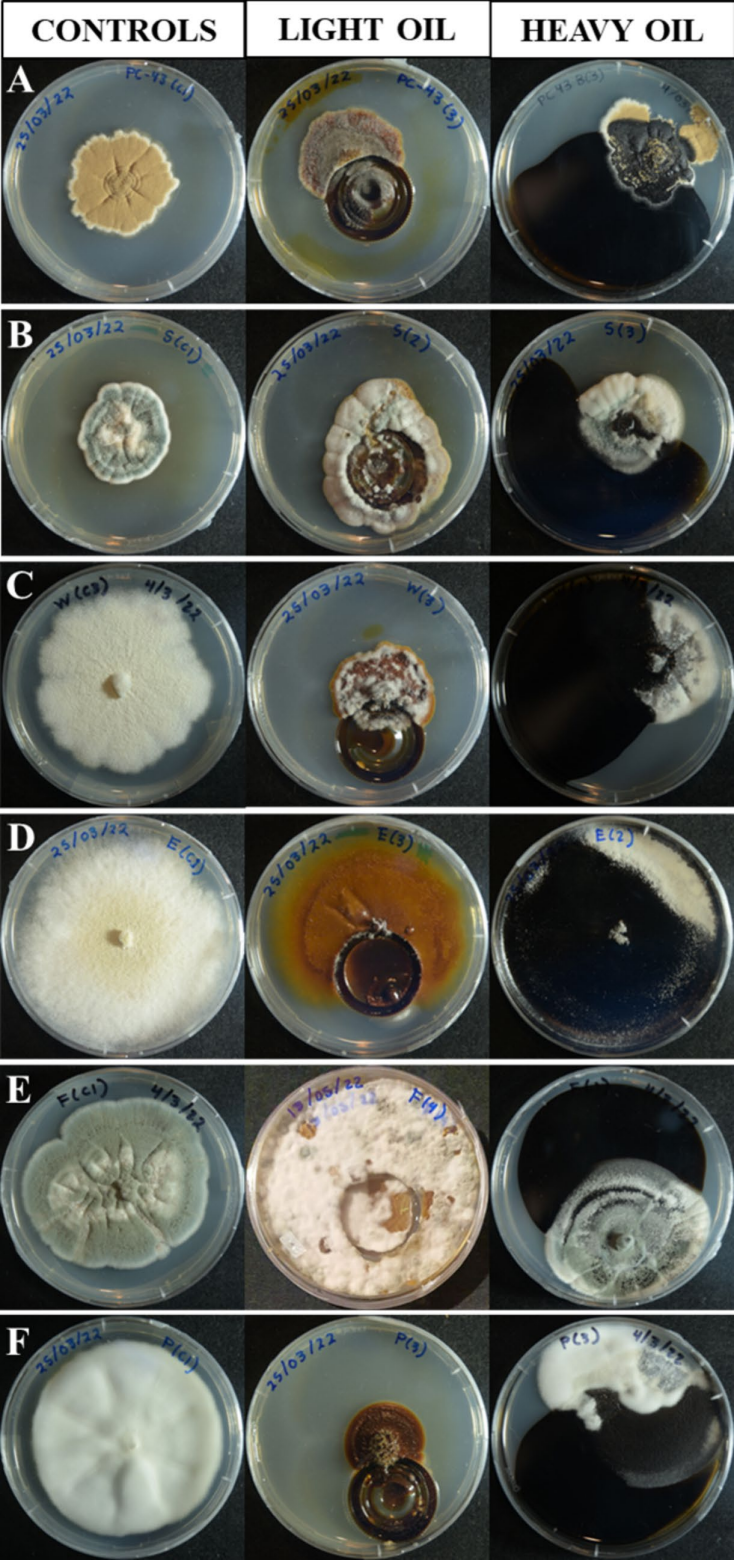
Growth of the isolates in the tolerance bioassay with LCO and HCO on day 21. **A**) *A. terreus.* **B**) *A. sydowii* **C**) *Aspergillus* sp. **D**) *P. miczynskii* (isolate E). **E**) *P. miczynskii* (isolate F). **F**) *P. dodgei*

**Fig. 2:**
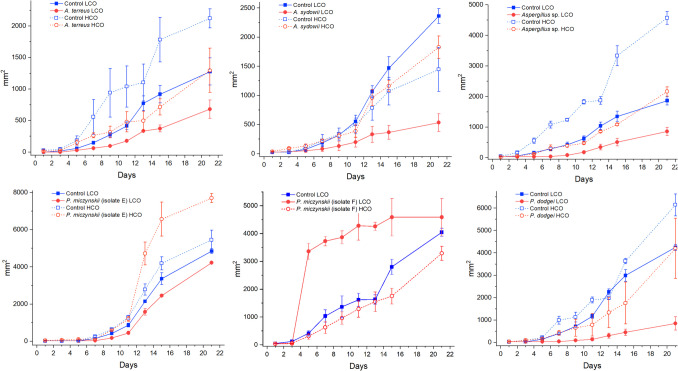
Fungal growth curves during the tolerance bioassay with LCO and HCO

All the tested isolates were able to grow in the presence of both types of crude oil in the tolerance bioassay (Fig. 1). However, responses differed among taxa. In the bioassay with LCO, all controls grew above 1250 mm^2^ at the end of the experiment, while most of the treatments grew between 530 and 860 mm^2^ (Fig. 2). Most of the controls grew more than the isolates exposed to the hydrocarbon except for *P. miczynskii* (isolate F), which grew 12% more in the presence of LCO, almost completely covering the Petri dish since day seven (Fig. 2). In contrast, controls never reached the edge of the Petri dish. In this bioassay, all the isolates, except for *A. sydowii* (Fig. 1), kept the radial growth pattern displayed by the controls. However, they displayed a brownish color on the LCO.

A similar pattern was observed in the bioassay with HCO since only a few isolates grew more in the experiment than in the controls: *A. sydowii* and *P. miczynskii* (isolate E) grew 21 and 29% more, respectively (Fig. 2). The last isolate reached the edge of the Petri dish at the end of the bioassay. Interestingly, the radial growth pattern of the isolates appeared altered by the HCO (Fig. 1), which may act as a barrier in some cases. Some parts of the mycelia seemed to be black and colored by hydrocarbons.

The statistical analysis of fungal growth during the tolerance bioassay revealed significant differences between treatments (*p* < 0.05), indicating differences between the controls devoid of oil and the experiments with oil. This suggests that the isolates exhibit different growth rates in the presence of petroleum, which may also vary depending on the viscosity and composition of the oil.

### Biodegradation bioassay

**Fig. 3:**
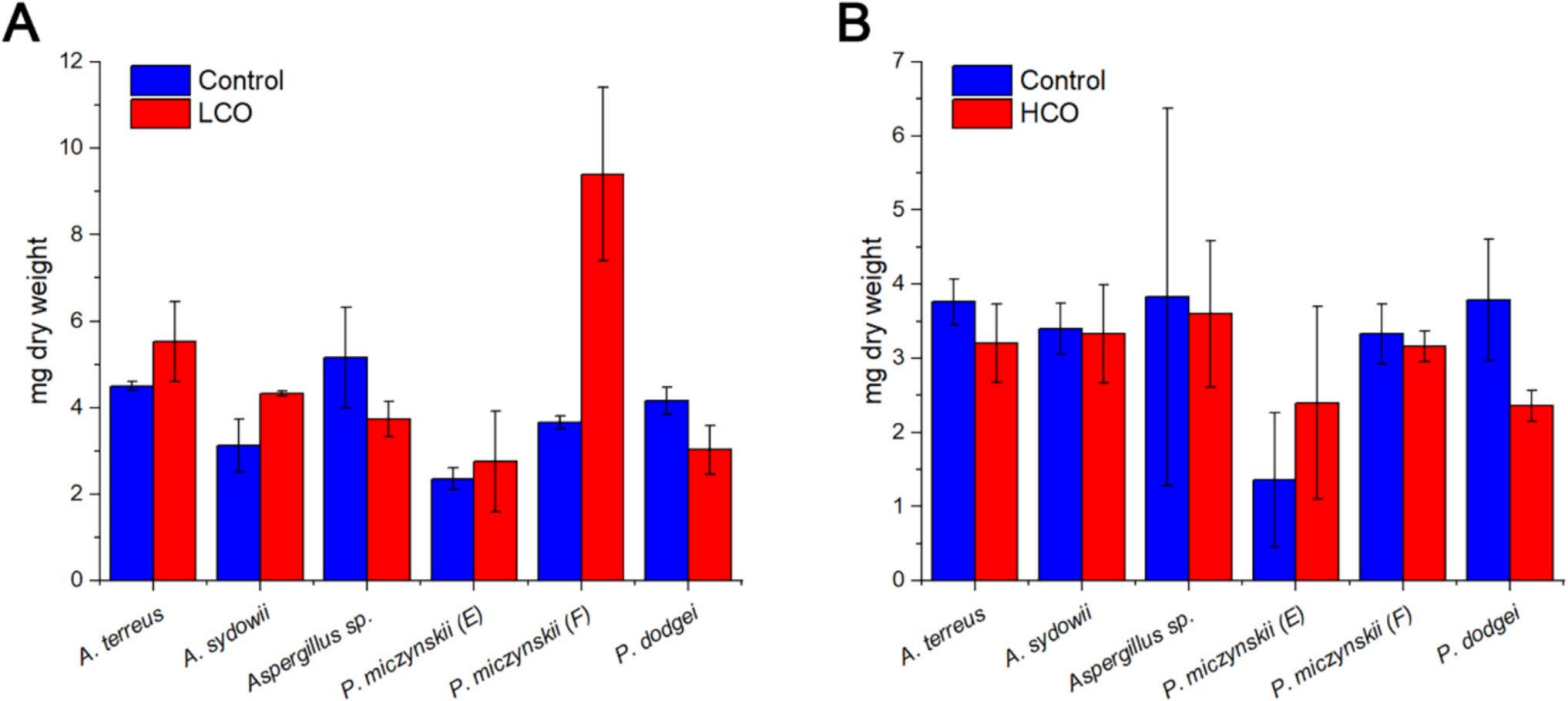
Growing rates (mg dry weight biomass) of the isolates in the controls and the biodegradation bioassay with LCO (**A**) and HCO (**B**)

All the isolates grew in the controls and in the presence of oil as a unique carbon source. However, they showed different growth rates between treatments (Fig. 3). Four isolates grew more in the presence of LCO as the sole carbon source than the controls, *P. miczynskii* (isolate F) being the most remarkable (with a growth rate of 157% above the controls), followed by *A. sydowii* (38%), *A. terreus* (23%), and *P. miczynskii* (isolate E) (15%) (Fig. 3A). In contrast, only *P. miczynskii* (isolate E) grew more using HCO as the sole carbon source (76%). At the same time, the rest of the isolates had higher dry biomass values in the controls, but were similar to those found in the treatments (Fig. 3B).

The PERMANOVA analysis performed on the fungal dry biomass obtained from the bioassay with LCO showed significant differences between treatments (*p* < 0.05) and isolates (*p* < 0.05). This hints that the growth of the isolates was different between the controls and the treatments since the average dry weight of the biomass was higher in most of the isolates exposed to oil,except for *Aspergillus* sp. and *P. dodgei*, where the growth was higher in the controls. The analysis of the differences between isolates showed that *P. miczynskii* (isolate F) and *A. terreus* differed from the rest of the strains since they had the highest biomass during the bioassay, 9.4 and 5.53 mg, respectively. In contrast, PERMANOVA of the bioassay with HCO showed no significant differences between treatments, only between isolates (*p* < 0.05), particularly between *A. sydowii* and *P. dodgei*.

### Isotopic analysis

The LCO had a *δ*^13^C value of −27.5‰ and a *δ*^15^N value of − 1.24‰ while the HCO showed a *δ*^13^C signature of − 27.36‰ and a *δ*^15^N of − 1.35‰. Concerning the isolates, the *δ*^13^C values of the controls varied between − 16.7 and − 12.3‰ (− 14.9 ± 1.08‰), while the *δ*^15^N signatures were from − 10.9 and 3‰ (− 3.8 ± 4.61‰) (Table 1). In the fungi exposed to light oil, the *δ*^13^C values were from − 24.8 to − 14.1 (− 16.8 ± 3.02‰). In contrast, the *δ*^15^N values varied between −6.2 to 4.8‰ (− 0.4 ± 3.5‰). In the fungi exposed to heavy oil, the *δ*^13^C values varied between −20.4‰ and − 13.7 (− 16.2 ± 1.51‰), while their *δ*^15^N values were from − 13.9 to 2.1‰ (− 5.4 ± 4.47‰) (Table 1).

**Table 1:** Carbon and nitrogen isotopic values of the controls and the isolates exposed to LCO and HCO as the unique carbon sources

Isolate	*δ*^13^C			*δ*^**15**^N		
	Control	LCO	HCO	Control	LCO	HCO
*Aspergillus terreus*	− 15.08	− 16.74	− 16.65	− 6.53	0.85	− 7.01
*Aspergillus sydowii*	− 14.74	− 15.59	− 15.63	− 5.18	0.89	− 6.7
*Aspergillus* sp.	− 14.89	− 15.83	− 15.88	− 4.36	− 1.82	− 5.3
*Penicillium miczynskii* (E)	− 16.24	− 18.74	− 17.42	1.22	− 0.23	− 2.75
*Penicillium miczynskii* (F)	− 14.3	− 19.18	− 16.11	− 4.18	− 1.64	− 7.22
*Penicillium dodgei*	− 14.74	− 14.87	− 15.22	− 1.95	− 0.55	− 3.3

**Fig. 4:**
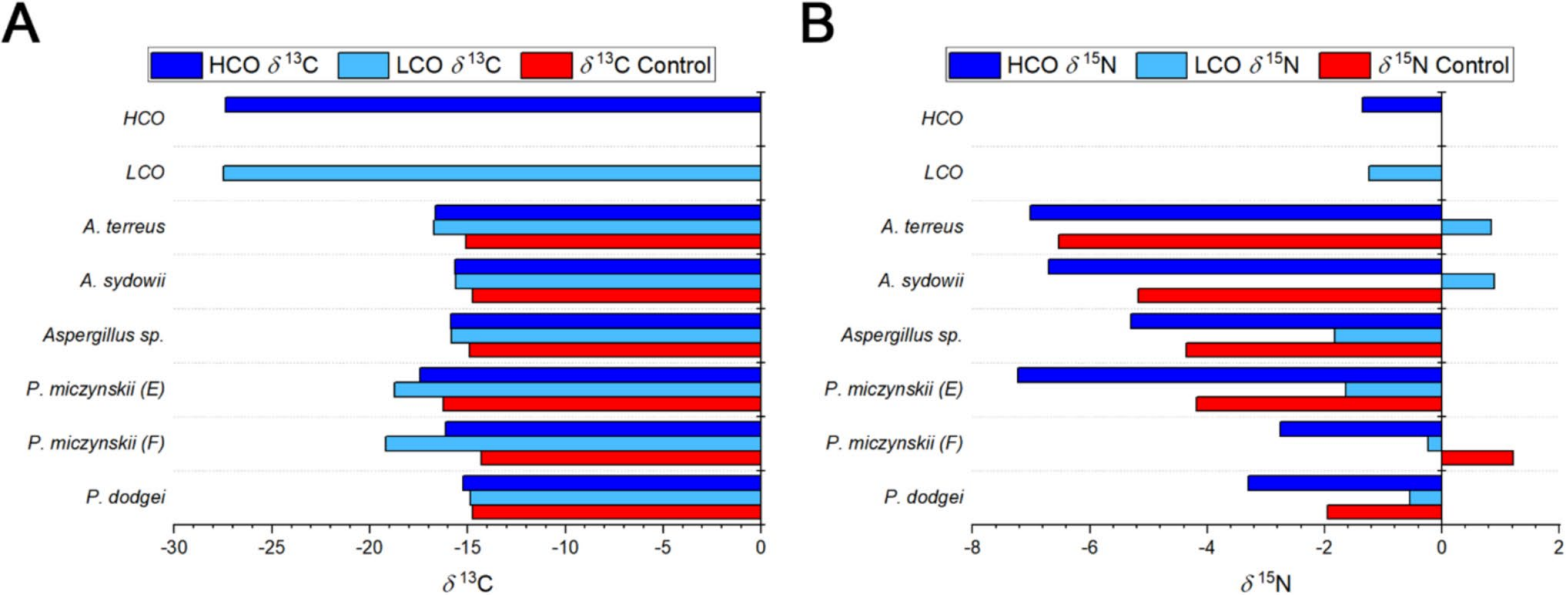
Comparison of the carbon (**A**) and nitrogen (**B**) stable isotopes between the LCO and HCO and the isolates exposed to them

It is worth noting that all *δ*^13^C values of the isolates were considerably enriched compared to both types of crude oil (Fig. 4A). Similarly, all the controls showed carbon isotopic signatures more enriched than the isolates exposed to crude oil (between 1.3‰ and 1.9‰) (Fig. 4A), which indicates that different carbon sources are being utilized. The *δ*^15^N values were more heterogeneous, and no clear pattern was observed. In the case of the strains exposed to LCO, both depleted and enriched values related to the hydrocarbon were noted. As for the isolates exposed to HCO, all had values lower than those of petroleum (Fig. 4B).

The PERMANOVA analysis on the *δ*^13^C values showed significant differences (*p* < 0.05) among the controls and the strains exposed to both types of oil. These differences suggest that the fungal strains assimilated different carbon sources under the three treatments. Similarly, the statistical analyses conducted on *δ*^15^N ratios displayed significant differences between treatments, specifically between the strains exposed to HCO and the controls and those exposed to LCO.

### Oil composition

#### Heavy crude oil

**Fig. 5:**
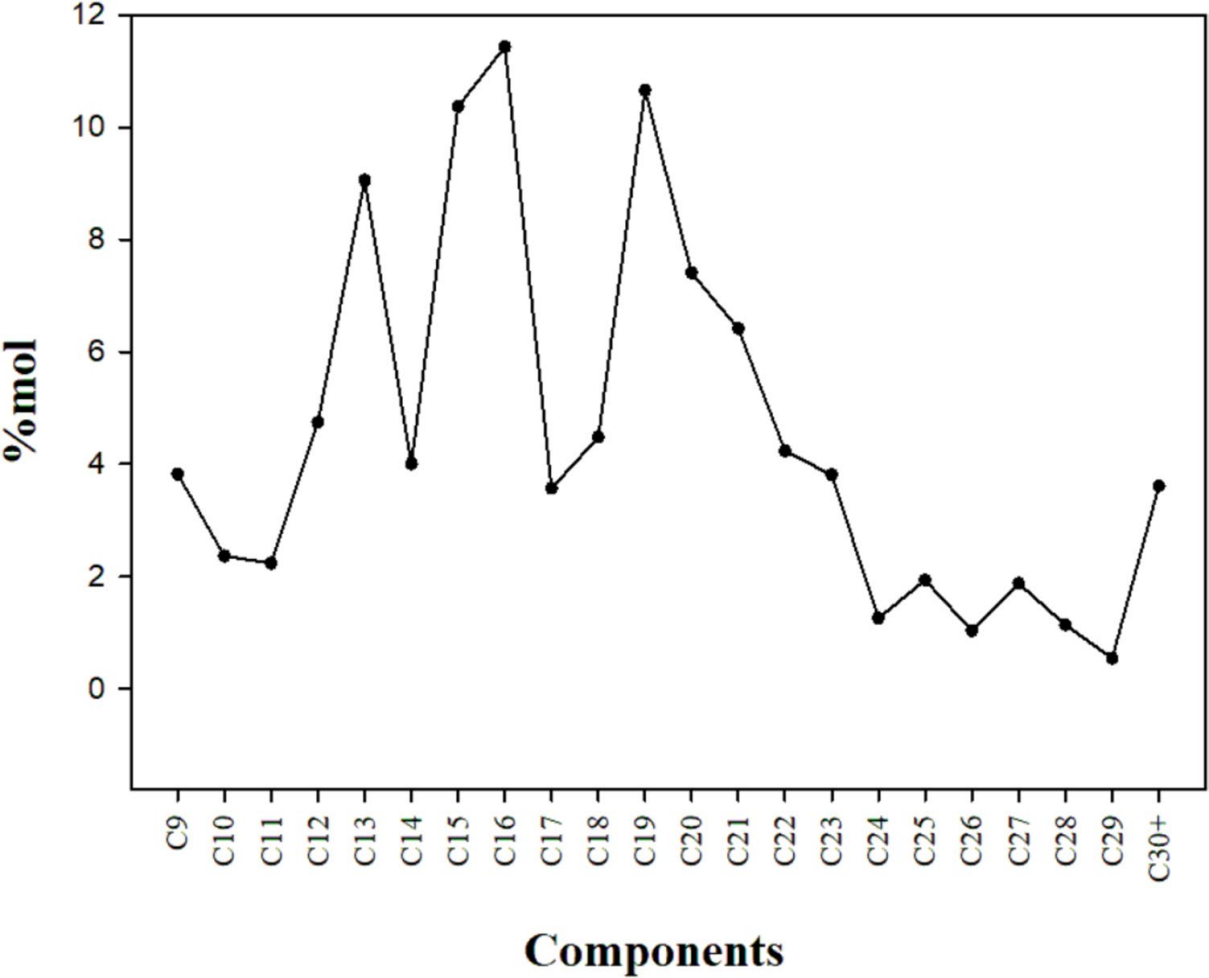
Hydrocarbon composition of the HCO

Compositional analysis of the original HCO showed that the interval of identified hydrocarbon components goes from C9 to C30 + (Fig. 5). The dominant components were mainly distributed in the range of C13-C21 and accounted for 67.4% of the oil´s global molar composition. The least abundant components were C24-C29 (Fig. 5), accounting for only 7.78.

### Light crude oil

**Fig. 6:**
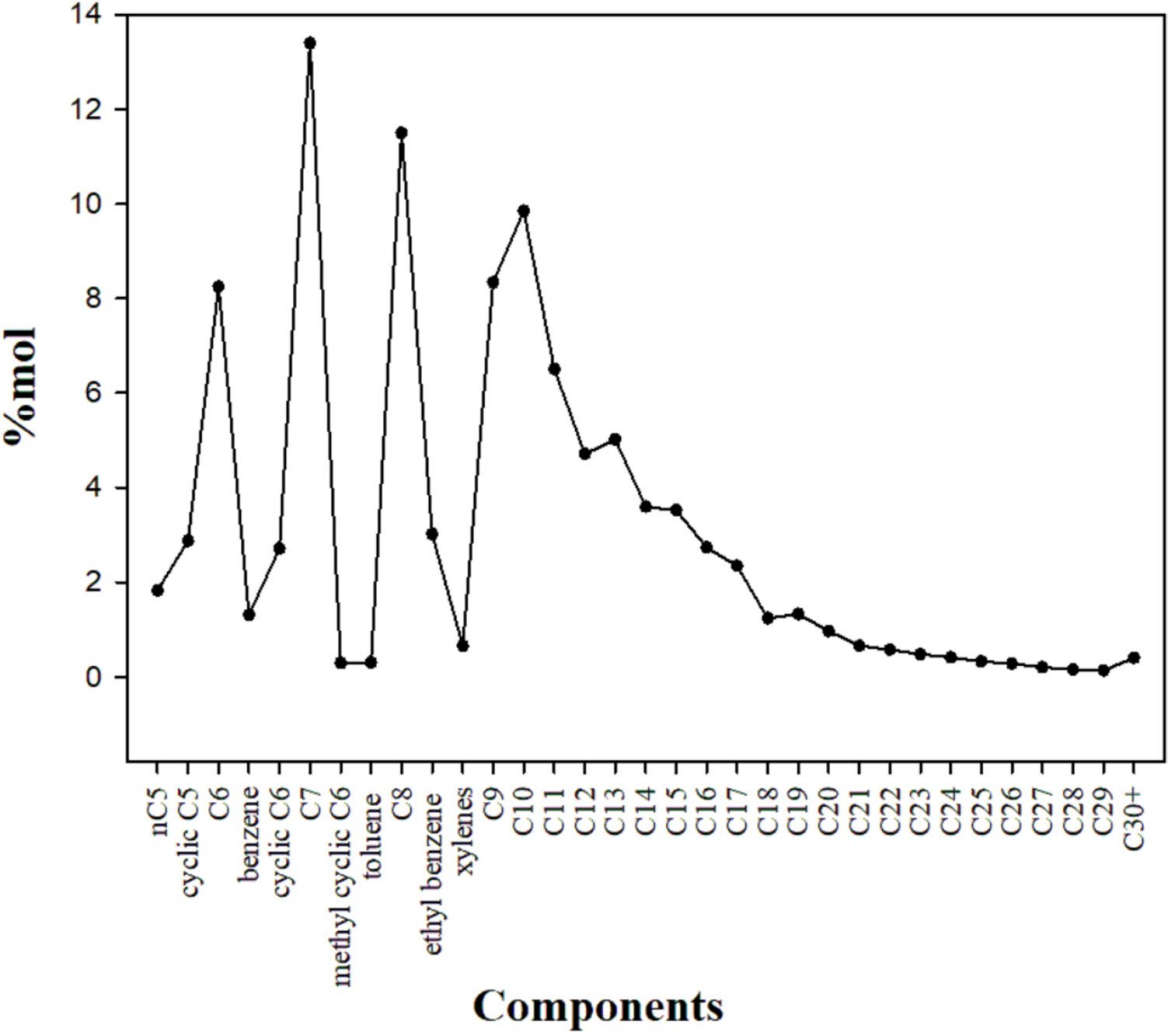
Hydrocarbon composition of the LCO

The compositional analysis of LCO indicated that the interval of identified hydrocarbons ranges from C5 to C30 +. The dominant components, accounting for 89.21% of the oil’s global molar composition, were mainly distributed between C6 and C13. In contrast, the less abundant components ranged from C18 to C30 + (Fig. 6), accounting for only 7.19%.

### Fungal activity on heavy oil

**Fig. 7:**
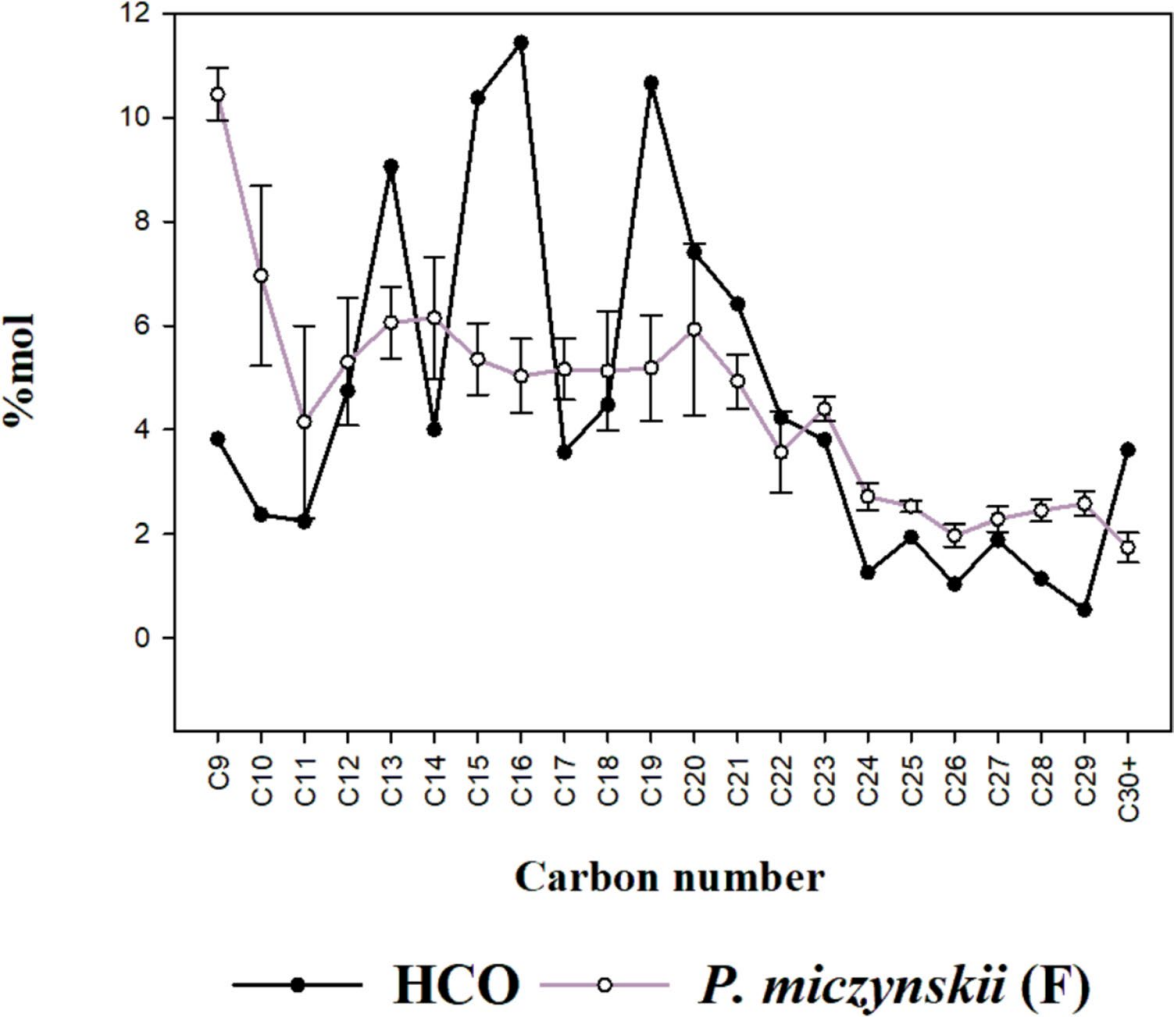
Comparison between the HCO composition before and after exposure to *P. miczynskii* (isolate F) activity

**Fig. 8:**
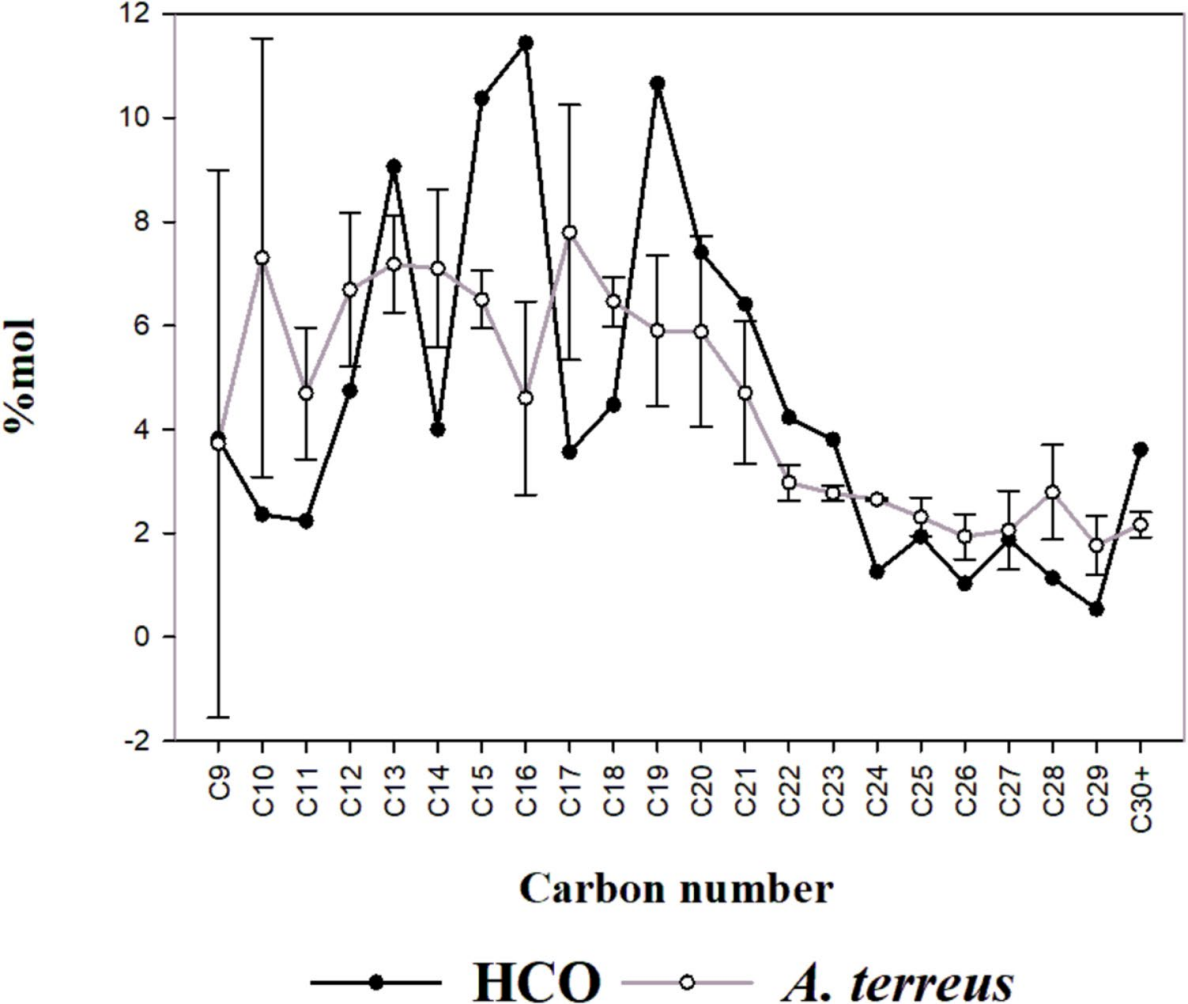
Comparison between the HCO composition before and after exposure to *A. terreus* activity

The HCO samples exposed to *P. miczynskii* (isolate F) and *A. terreus* showed substantial compositional changes compared to the petroleum not exposed to biological activity (Figs. 7–8). Interestingly, the degree of degradation of hydrocarbons in the samples exposed to different strains was very similar.

The C30 + content of the oil molar fraction decreased by 53% and 42% in the presence of *P. miczynskii* (isolate F) and *A. terreus,* respectively. In contrast, C-24-C29 components doubled after exposure to both strains. The C13, C15, C16, and C19-C22 hydrocarbons accounted for more than 60% of the HCO´s molar fraction and were depleted in the samples exposed to fungal activity (Figs. 7–8), which was 36% in the presence of *P. miczynskii* (isolate F) and 38% with *A terreus*. Finally, lighter hydrocarbons C9-C11 in the oil exposed to both strains increased twice their percentage. The fungal activity changed the sequencing proportions of components, with heavy and medium-weight compounds decreasing while the immediately lighter ones increased, suggesting selective hydrocarbon degradation.

## Discussion

### Tolerance and biodegradation bioassays

Microscopic fungi from extreme ecosystems have been proven to possess unique biodegradation capabilities due to their adaptation to achieve optimal growth and reproduction under extreme conditions (Cerniglia and Sutherland 2010; Rampelotto 2013). Moreover, several marine-derived fungi have been recognized for their strong capabilities to degrade crude oil and individual hydrocarbons (Kirk and Gordon 1988; Passarini et al. 2011). However, to our knowledge, no previous reports on crude oil-degrading fungi from hydrothermal vents are available. Herein, we demonstrated the ability of six fungal isolates obtained from deep-sea vents to grow in the presence of LCO and HCO, which suggests their tolerance towards simple and complex hydrocarbons. This may be related to the close contact of these fungi with hydrocarbons in their natural environment since Paduan et al. (2018) reported the occurrence of hydrocarbons ranging from methane to heptane in Pescadero Basin vent sediments. It is worth mentioning that both *A. terreus* and *P. miczynskii* (isolate F) had been isolated from Pescadero Basin sediments with visible oil drops and an intense oil odor (Salcedo et al. 2023).

The fungal strains displayed different growth patterns when exposed to different oils in the tolerance bioassay, which showed inter- and intraspecific variability in their capacities to use the same carbon source. Bovio et al. (2017) and Maamar et al. (2020) observed that several isolates of the same species exhibited different performances in the presence of various crude oils, suggesting intraspecific variability in the biological response to petroleum.

All the isolates appeared to use crude oil as the sole carbon source in the biodegradation bioassay. However, most fungal strains proved more efficient using light oil hydrocarbons since four strains had higher growth rates than the unexposed controls. In contrast, only *P. miczynskii* (isolate E) could efficiently use carbon from HCO to grow, which suggests its potential ability to degrade heavier or recalcitrant compounds. This indicates that HCO´s degradation efficiency was lower than LCO´s, which may be attributed to the complex mixture of crude oil consisting of insoluble compounds such as n-alkanes, resins, and asphalts (Al-Hawash et al. 2018). The chemical composition of different hydrocarbons is essential in determining fungi's ability to produce biomass (Prenafeta-Boldú et al. 2018; Al-Otibi et al. 2022).

*Penicillium miczynskii* (isolate F) exhibited the highest growth among all tested isolates when using LCO as a unique carbon source, reaching 157% of its biomass production. Previous studies have shown the hydrocarbon degradation capabilities of this species. *P. miczynskii* (st.1) was able to degrade 85% of petroleum hydrocarbons in contaminated soil, being classified as a species with high hydrocarbon-oxidizing activity. On the other hand, *P. miczynskii* (st.2) achieved a 73% degradation rate, being labeled as a strain with medium hydrocarbon-oxidizing activity (Myazin et al. 2021). These findings suggest the potential of this species in the bioremediation of contaminated soils, particularly in extreme environments.

Concerning *A. terreus*, our results agree with former findings on its hydrocarbon degradation capabilities. For instance, Bovio et al. (2017) observed that this fungus uses crude oil as a source of nourishment and suggested that it has a strong bioremediation potential. Several authors demonstrated that this species has a solid capability to degrade short-chain *n*-alkanes (< C18), long-chain *n*-alkanes (C19-C36), pristane, phytane, and PAHs, with a preferential degradation of short-chain alkanes (Colombo et al. 1996; Elshafie et al. 2007; Lotfinasabal et al. 2012; Simister et al. 2015; Bovio et al. 2017).

### Isotopic signatures

#### Isotopes in crude oil

The carbon isotopic signatures of both oils were very similar: ‒27.5‰ in the LCO and ‒27.3‰ in the HCO. These values plot in the range of worldwide occurrences (‒26 to ‒28‰) (Hirner and Lyon 1989). Furthermore, oil from the Gulf of Mexico has been shown to have a consistently stable carbon isotope signature of around ‒27.3‰ (Graham et al. 2010; Macko et al. 1981). In the case of the nitrogen isotopic ratios, the values of the LCO (‒1.24‰) and the HCO (‒1.35‰) were depleted in comparison to the values most frequently found in crude oils (between 0.74‰ and 3.4‰) (Grizzle et al. 1979; Hahn-Weinheimer and Hirner 1980; Hirner and Lyon 1989).

#### Fungal carbon isotopes

To the best of our knowledge, this is the first research addressing the isotopic values of fungi exposed to crude oil as a sole carbon source. Some authors have observed a clear pattern where fungal isotopic carbon ratios closely follow those of their carbon sources or are enriched. Hence, *δ*^13^C measurements can be used to establish the potential carbon sources fungi use (Hobbie 2005).

Saprotrophic fungi are enriched by 1–3‰ compared to wood or agar–agar mixtures where they were cultivated (Hobbie 2005), whereas ectomycorrhizal fungi are enriched by 1–5‰ in relation to their host (Hobbie et al. 1999; Högberg et al. 1999; Kohzu et al. 1999). Remarkably, pelagic fungi have shown an enrichment of 2.5‰ relative to their carbon source (Abraham and Hesse 2003), while hydrothermal vent fungi have exhibited an enrichment ranging from 4.52‰ to 12.6‰ concerning their provenance sediments (Salcedo et al. 2023). Notably, some isolates are the same as those analyzed here (*A. terreus* and *P. miczynskii* (isolate F). A similar trend was seen in the isolates used in this study since all of them had *δ*^13^C values enriched by 10.68 ± 1.76‰ relative to the LCO and by 11.21 ± 0.79‰ concerning the HCO (Fig. 4). Perhaps the degradation of certain hydrocarbons (naturally present in their environment and the crude oils) results in high fractionation patterns. This can be especially true for *A. terreus* and *P. miczynskii* (isolate F) from the Pescadero Basin vents since both were isolated from an oily sediment sample (Salcedo et al. 2023).

The PERMANOVA analysis on fungal carbon isotopic ratios displayed significant differences between treatments (controls, LCO, and HCO), so the three groups of isolates seem to use different carbon sources. It is worth mentioning that the isotopic ratios of the petroleum correspond to the bulk crude oil, including all its fractions, so the different isotopic values between the isolates suggest that they may be selectively using certain hydrocarbons.

#### Fungal nitrogen isotopes

It is thought that the nitrogen isotopic signature of fungal biomass can provide information on the substrates used by fungi (Constantini et al. 2014). Most isolates exposed to both crude oils had depleted values relative to the petroleum, with few exceptions (*P. dodgei*, *P. miczynskii* (F), *A. terreus*, and *A. sydowii*) in LCO. Some authors have reported that the fungal *δ*^15^N values are generally depleted with respect to their growth medium or nitrogen source (see Henn and Chapela 2004; Semenina and Tiunov 2010; Abraham and Hesse 2003). Salcedo et al. (2023) found that ten isolates from hydrothermal vents showed lower *δ*^15^N values than their provenance sediments, displaying fractionation patterns between 1.05 ± 3.19‰ and 8.15 ± 2.76‰.

The PERMANOVA analyses on nitrogen isotopic ratios suggested that the strains exposed to HCO differed from those exposed to LCO. There is a pattern where the isolates exposed to the LCO fractionate against the light isotope. Consequently, their biomass has more heavy isotopes, resulting in isotopically enriched values relative to the oil. In contrast, the isolates exposed to the HCO seem to discriminate against the heavy isotope and accumulate more of the light isotope in their biomass, resulting in more depleted values. The discrimination against the heavy isotope during uptake can influence the *δ*^15^N values (Handley and Raven 1992; Fogel and Cifuentes 1993). Enzymatic reactions fractionate to a greater or lesser extent against heavier isotopes (Hoering 1955). Constantini et al. (2014) suggested that fungi lose N^14^ during metabolic turnover, which varies in different degrees, depending on biomass accrual rate and strain-specific physiological differences. Henn and Chapela (2004) observed a preferential incorporation of the light isotope into fungal biomass, although it seems not to be a rule. The joint analysis of *δ*^13^C and *δ*^15^N values suggests that the fungal isotopic signatures result from both energy sources (individual hydrocarbons) and different fractionation patterns driven by the activation of diverse metabolic pathways or enzymes.

#### Oil degradation by fungi

Compositional changes occurred in the residual oil relative to the original composition. The increased ratio in medium-chain hydrocarbons such as C9, C10, C11, C12, C14, C17, and C18 after being exposed to the activity of both isolates indicated the emergence of lighter hydrocarbons as a result of the biodegradation of long-chain hydrocarbons from C19 to C23. The percentage of C19 to C22 decreased in both isolates, suggesting that *P. miczynskii* (isolate F) and *A. terreus* can use these hydrocarbons as a carbon source. Interestingly, long-chain hydrocarbons >  30 C also diminished in the presence of both isolates, while C24 to C29 increased. These results reveal that both fungal strains can selectively degrade long-chain hydrocarbons (C > 18). Bovio et al. (2017) pointed out that *A. terreus* uses crude oil as a nourishment source, which has degradation potential for C14 and C24 compounds. Different factors control the microbial biodegradation rate of hydrocarbons, such as their type, availability, length, volatilization, and solubility (Chandra et al. 2013; Lawniczak et al. 2020; Al-Otibi et al. 2022). Some light hydrocarbons could be lost due to the conditions of the experiments since the biodegradation bioassay was not conducted on a completely closed system**.** However, light hydrocarbons increased their ratios, which could only result from fungal activity.

## Conclusions

All the hydrothermal vents fungal isolates tested showed tolerance to LCO and HCO exposure, with different growth patterns regarding the controls devoid of hydrocarbons. Similarly, all the isolates were able to use crude oil as a sole carbon source. However, *A. terreus*, *A. sydowii*, *Aspergillus* sp., *P. miczynskii* (isolates E and F) showed enhanced growth in the presence of oil, highlighting their biodegradation potential. The enriched *δ*^13^C values indicated that the fungal biomass assimilated carbon from crude oil and that the fractionation was considerably higher than fungi from other environments. This high fractionation (> 10‰) could result from enzymatic mechanisms inherent to vent fungi and the assimilation of individual hydrocarbons from the oil. Additional information could be obtained by analyzing the carbon isotopic composition of the oil residues after harvesting the biomass derived from the biodegradation bioassay. The heterogeneity in the *δ*^15^N values reflects intraspecific variability or selectivity in using nitrogen sources. The analysis of the oil composition exposed to fungal activity showed that *P. miczynskii* (isolate F) and *A. terreus* could selectively degrade medium and large-chain hydrocarbons. However, these were not the most abundant compounds in the oils tested. This again highlights the fungal potential to degrade complex/heavy hydrocarbons and their prospective for bioremediation applications in oil-contaminated environments. Given all the analyses conducted in this study, *P. miczynskii* (isolate F) was the most promising candidate for an effective bioremediation process.

Deep-sea fungi offer promising bioremediation applications due to their unique enzymatic capabilities in extreme conditions, yet practical considerations like scalability, culturing difficulty, and biosafety in non-native environments pose significant limitations. Future research should focus on genetic engineering to enhance their robustness and developing in situ bioremediation techniques to overcome these constraints (Harms et al. 2011). Particularly, metabolomic analyses are necessary to verify the ability to degrade crude oil in nature of our isolates. Additionally, bioassays with individual hydrocarbons and their respective isotopic measurements should be conducted to determine the assimilation of oil derivatives into fungal tissues and more precise fractionation patterns.

## Data Availability

All data supporting the findings of this study are available within the paper. Additional information is available upon request.
